# Internal Adaptation of Composite Fillings Made Using Universal Adhesives—A Micro-Computed Tomography Analysis

**DOI:** 10.3390/ma17030636

**Published:** 2024-01-28

**Authors:** Kinga Kaczor-Wiankowska, Adam K. Puszkarz, Mirona Palczewska-Komsa, Sebastian Lipa, Michał Krasowski, Jerzy Sokołowski, Katarzyna Lewusz-Butkiewicz, Katarzyna Ulacha, Alicja Nowicka

**Affiliations:** 1 Department of Conservative Dentistry and Endodontics, Pomeranian Medical University in Szczecin, 72 Powstancow Wielkopolskich Str., 70-111 Szczecin, Poland; 2 Division of Materials Science, Commodity Science and Textile Metrology, Textile Institute, Faculty of Material Technologies and Textile Design, Lodz University of Technology, 116 Zeromskiego Str., 90-924 Lodz, Poland; 3 Department of Dental Prosthetics, Pomeranian Medical University in Szczecin, 72 Powstancow Wielkopolskich Str., 70-111 Szczecin, Poland; 4 Institute of Materials Science and Engineering, Lodz University of Technology, 1/15 Stefanowskiego Str., 90-924 Lodz, Poland; 5 Material Science Laboratory, Medical University of Lodz, 251 Pomorska Str., 92-213 Lodz, Poland; 6 Department of General Dentistry, Medical University of Lodz, Pomorska 251 Str., 92-213 Lodz, Poland

**Keywords:** marginal integrity, self-etch, etch and rinse, adhesive, composite filling, micro-CT

## Abstract

This study aimed to evaluate internal tooth–filling interfaces of composite fillings made using universal adhesives using micro-computed tomography (µCT). Sixty class V cavities were randomly assigned into six groups: Peak Universal etch and rinse (PER), Peak Universal self-etch (PSE), Adhese Universal etch and rinse (AER), and Adhese Universal self-etch (ASE). Two further adhesives considered gold standards were used as control groups: OptiBond FL (OER) for the etch and rinse technique and Clearfil SE for the self-etch technique (CSE). All teeth were subjected to thermomechanical loading and four-year water storage. Next, they were analyzed using µCT to investigate the internal tooth–filling interfaces. The proportions between the gap volume (GV) at the tooth–filling interface and the volume of applied composite filling (FV), between the gap and cavity volumes (CV), and between the gap volumes at the tooth–filling interface of the external (EGV) and internal (IGV) parts were calculated. Adhese Universal achieved the significantly lowest gap-to-filling- and gap-to-cavity-volume ratios for both types of etching techniques comparing to those of the Peak Universal and control groups. Significant differences between the gaps in external and internal parts of the tooth–filling interface were only noted in the control groups. Internal gap formation and development at the tooth–filling interface depend on the material as well as the type of its application.

## 1. Introduction

Modern classifications divide dental adhesives according to their generation or by their procedure with the smear layer [1]. Adhesives that remove the smear layer and open dental tubules using phosphoric acid etching are known as etch and rinse adhesives (ER). Adhesives that do not use a separate etching step are known as self-etch adhesives (SE) [1]. SE techniques are easier to use and more conservative for dentin; however, the use of phosphoric acid helps prepare the enamel for the adhesive procedure. To combine the advantageous features of both types of adhesives and to simplify and introduce a less sensitive technique, new universal adhesives have been proposed [2,3].

Improving the adaptation and longevity of composite fillings are among the main challenges of modern adhesive dentistry and laboratory dental research. Gaps (free space), especially internal, at the tooth–filling interface lead to bacterial and water penetration verifiable in in vitro studies, which may clinically induce discoloration, postoperative sensitivity, secondary caries, pulp inflammation, and composite filling loss [4,5,6]. Previous studies have proven that the quality of the tooth–filling interface depends on procedural and material factors such as the composition and type of the adhesive system, etching approach, type and shrinkage stress of the restorative material, size and type of the cavity, and the insertion and polymerization technique [7,8,9,10]. Some components of adhesive systems, such as bisphenol A-glycidyl methacrylate or 2-hydroxyethyl methacrylate (HEMA), can be cytotoxic to the pulp [11]. These cytotoxic effects depend on the amount of free, unpolymerized resin monomers and the permeability and thickness of dentin, especially of the pulp wall. The monomers do not have to be in direct contact with the pulp but can diffuse through the dentinal tubules in concentrations and induce a toxic effect on it [12,13]. Furthermore, unpolymerized monomers leach out and expose collagen fibrils which will hydrolytically degrade. It has been associated with the degradation of hydrophilic resin components (e.g., HEMA), leading to damage of the hybrid layer and loss of bonding durability of adhesives over time [4,14,15].

Aging factors, such as water storage, mechanical loads, and temperature changes, have a negative effect on the tooth–filling interface and lead to the formatting or development of internal gaps [4,5,16,17]. The tooth–filling interface mainly degrades chemically or mechanically. It is exposed to water and enzymes at first, and then, enzymes released from the dentin matrix might lead to resin or collagen hydrolysis and degradation of adhesives and composite resin components [14,17]. Moreover, water can cause “plasticization” of resin, which refers to swelling and the reduction of the frictional forces of the polymer chains, causing a decrease in the mechanical properties of the polymer matrix [18]. Further, mechanical loads affect the tooth–filling interface during each chewing cycle. When occlusal stress exceeds the material fracture strength, it may lead to crack initiation or propagation, which affects the structural integrity of the filling [17]. In addition, temperature changes may induce repetitive contraction and expansion stress at the tooth–filling interface and enlarge existing gaps, which leads to increased water absorption [19].

The quality of an internal tooth–filling interface can be assessed using destructive or non-destructive methods. Destructive methods use a tracer or dye and damage the sample by cutting it into sections to visualize the extent of staining along the tooth–filling interface using methods such as light microscopy, scanning electron microscopy (SEM), or optical microscopy [20,21]. Exemplary non-destructive methods are the replica technique in combination with SEM for external marginal adaptation [7,22] or X-ray micro-computed tomography (µCT) analysis [21,23]. This three-dimensional (3D) method enables the detection of all internal gaps and irregularities as well as their changes over time. Moreover, it allows for the analysis of the internal structure of restorative materials or tooth structures with a high accuracy and spatial resolution [24,25].

The purpose of this study was to use µCT to evaluate the internal tooth–filling interface of composite fillings made using universal adhesives applied with different etching modes after thermomechanical loading (TML) and four-year water storage in non-carious cervical lesions (NCCLs). The first null hypothesis was that there are no differences in the internal tooth–filling interface of both universal adhesives, Adhese Universal and Peak Universal. The second null hypothesis was that the etching technique does not influence the long-term effectiveness of universal adhesives, and the third null hypothesis was that gaps are mostly located at the external parts of the tooth–filling interface.

## 2. Materials and Methods

This study was approved by the local Ethics Committee of the Pomeranian Medical University, Szczecin, Poland (approval number KB-0012/82/11/2014). Caries-free human third molars, extracted for orthodontic reasons, were used in this study. The teeth were disinfected in an aqueous solution of 0.5% chloramine-T (Chempur, Piekary Śląskie, Poland) and used a maximum of six months after extraction. One operator (K.K-W.) prepared standardized class V cavities on the buccal and lingual surfaces of each tooth using a round diamond bur (Edenta-801.314.012, Edenta AG, St Gallen, Switzerland). The burs were changed every five preparations. The cavities were 4 mm in diameter (2 mm above and below the cement–enamel junction) and 2 mm deep (in the middle part of cavity, controlled by a caliper). The enamel bevels and finishing preparations were carried out using fine-grained diamond burs (Edenta-862.204.012, Edenta AG, Sankt Gallen, Switzerland). The teeth were randomly distributed into six groups (n = 10), according to the adhesive and etching approaches used. Two universal adhesives, Peak Universal (Ultradent, South Jordan, UT, USA) and Adhese Universal (Ivoclar Vivadent; Schaan, Lichtenstein) were applied with two etching modes, respectively, as the experimental groups: Peak Universal etch and rinse (PER), Peak Universal self-etch (PSE), Adhese Universal etch and rinse (AER), and Adhese Universal self-etch (ASE). Two further adhesives considered gold standards were used as control groups: OptiBond FL (OER; Kerr, Orange, CA, USA) was used as a control group for the ER group, and Clearfil SE (CSE; Kuraray Noritake Dental; Tokyo, Japan) was used as a control group for the SE group. The compositions, manufacturers, batch numbers, etching techniques of each adhesive system, composite resins, and their chosen features are shown in Table 1.

**Figure 1:**
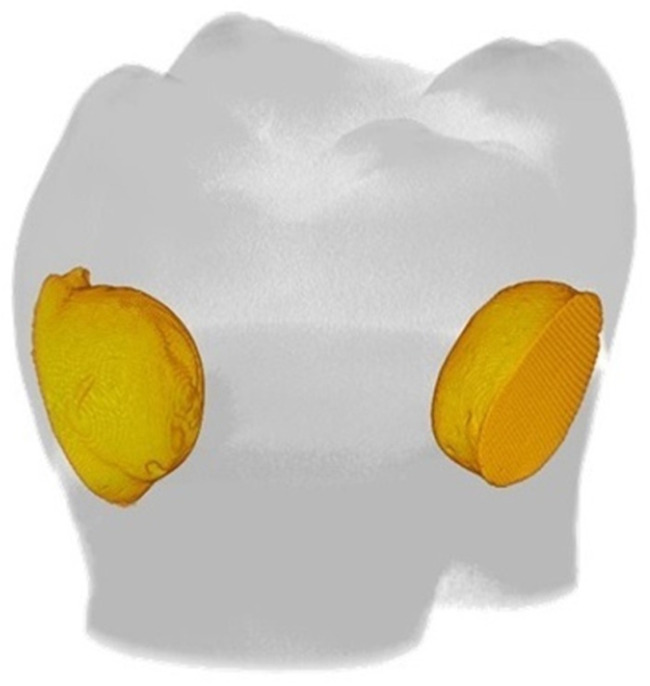
Representative tooth with two prepared fillings.

Next, the cavities were filled with two increments of composite resin material from the same manufacturer as the adhesive according to the manufacturer’s instructions and polymerized (Demi Plus, Kerr, Orange, CA, USA). Composite filling was applied in comparable external conditions (e.g., temperature and humidity) by one operator (K.K-W). Immediately after the procedure, the fillings were finished with a flexible disc (Soflex, 3M ESPE, Chicago, IL, USA). An exemplary tooth with two fillings is shown in Figure 1.

Immediately after restoration, all teeth underwent TML. Thermal cycling consisted of 3000 cycles in water at temperatures of 5 and 55 °C with a dwell time of 20 s in each temperature bath and a transfer time of 13 s (SD Mechatronik GmbH, Feldkirchen-Westerham, Germany). Mechanical loading was performed over 100,000 cycles with a load of 110 N at a frequency of 2 Hz using a rounded tip as an antagonist (Walter + Bai Dynamic Testing Systems LFV-50 kN, Walter + Bai AG, Löhningen, Switzerland). Then, long-term water storage in distilled water with a thermostat (TK-2, CABROLAB ELECTRONIC, Warsaw, Poland) took place for 4 years at 37 °C.

**Figure 2:**
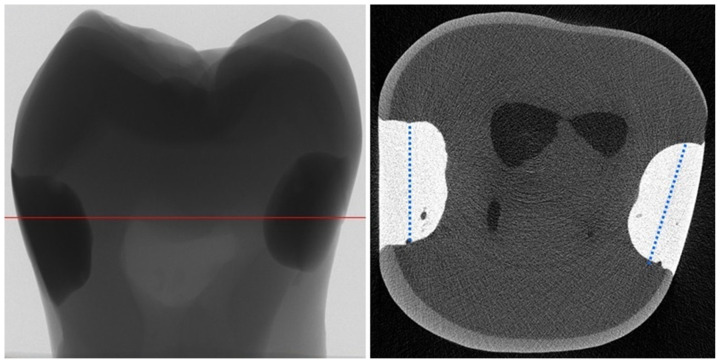
Division scheme of the tooth–filling interface into external and internal parts (blue lines divided fillings into two parts).

µCT scanning was performed using a SkyScan 1272 scanner (Bruker Corporation, Kontich, Belgium). The acquisition parameters were as follows: voltage, 90 kV; current, 111 μA; filter material, Al 0.5 + Cu 0.038; voxel size, 4.7 µm; rotation step and angle, 0.5°; exposure time, 1306 ms; resolution, 4032 × 2688 pixels. Three-dimensional reconstructions of the tooth fillings were obtained using the NRecon 1.7.4.2 and CTvox 3.3.0 r1403 software (Bruker, Billerica, MA, USA). Radiolucent space between tooth tissues and filling material was analyzed. Geometric parameters were calculated using the CTAn 1.17.7.2+ software (Bruker). The following parameters were quantified: GV—total volume of the gap (radiolucent; free space) at the tooth–filling interface (mm^3^) without including potential voids inside the composite material; FV—composite filling volume (mm^3^); and CV—cavity volume (mm^3^) defined as GV + FV. Similar to other research [25,28], the proportions between GV and FV (GV/FV ratio (%)) and between GV and CV (GV/CV ratio (%)) were calculated. The GV/FV and GV/CV ratios were introduced to make samples comparable between the different groups, despite differences in cavity size. At the end, researchers introduced their own analysis of the tooth–filling interface: a plane was drawn, dividing the filling into two parts, external—from the outside to the center of the filling—and internal—from the center of the filling to the pulp wall (Figure 2).

The gap volumes at the tooth–filling interface of the external part and internal part (in mm^3^) were calculated.

The Kolmogorov–Smirnov test revealed a non-normal data distribution in most groups; therefore, nonparametric tests (Kruskal–Wallis test and Mann–Whitney U test) were used for the analyses. The Kruskal–Wallis test determines whether the medians of groups are significantly different, but it does not show where the differences exist. The Mann–Whitney test was used to compare two groups. The significance threshold was set at *p* = 0.05.

## 3. Results

### 3.1. Gap Volume to Filling Volume (GV/FV) and Gap Volume to Cavity Volume (GV/CV) Ratios

Adhese Universal achieved the significantly lower GV/FV and GV/CV ratios for both types of etching techniques (ER: 1.9% and 1.8%, respectively; SE: 1.5% and 1.5%, respectively) than the Peak Universal groups. There was a significant difference between the Adhese Universal etch and rinse and Adhese Universal self-etch groups (GV/FV—*p* = 0.0292; GV/CV—*p* = 0.0382). Both etching techniques resulted in significantly lower GV/FV (ASE—*p* = 0.0017; ATE—*p* = 0.0011) and GV/CV (ASE—*p* = 0.0014; ATE—*p* = 0.0011) ratios than those in control groups.

Regarding Peak Universal, the SE technique resulted in lower GV/FV (2.4%) and GV/CV (2.4%) ratios than the ER technique, but the difference was not significant. Compared to the control group, only the PSE group had significantly lower GV/FV (*p* = 0.019) and GV/CV (*p* = 0.017) ratios. The PER group had higher GV/FV (3.1%) and GV/CV (3.0%) ratios than the OER group, but not significantly. The OptiBond FL control group had non-significantly lower GV/FV and GV/CV ratios than the Clearfil SE control group. Exact volumes, standard deviations, and ratios of all groups are presented in Table 2.

**Figure 3:**
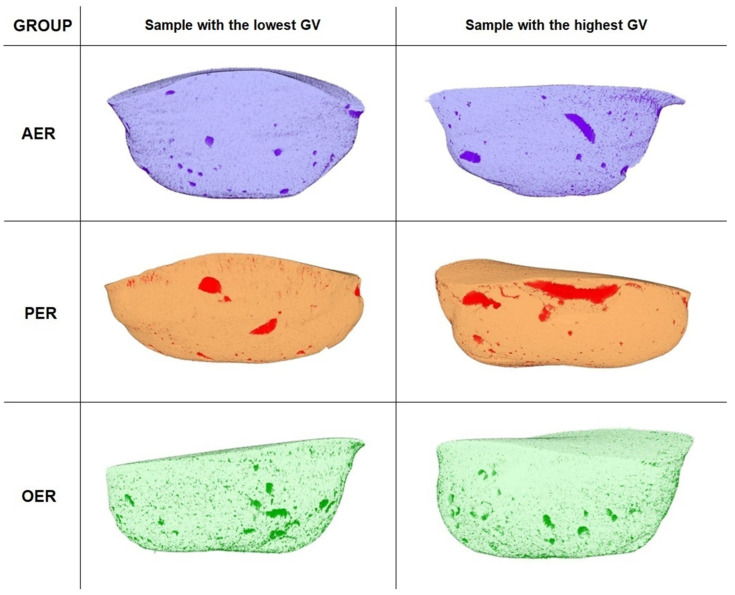
Three-dimensional reconstructions of samples with the highest and the lowest external gap volume values from the etch and rinse groups (free space marked by the most intense color of the reconstruction).

**Figure 4:**
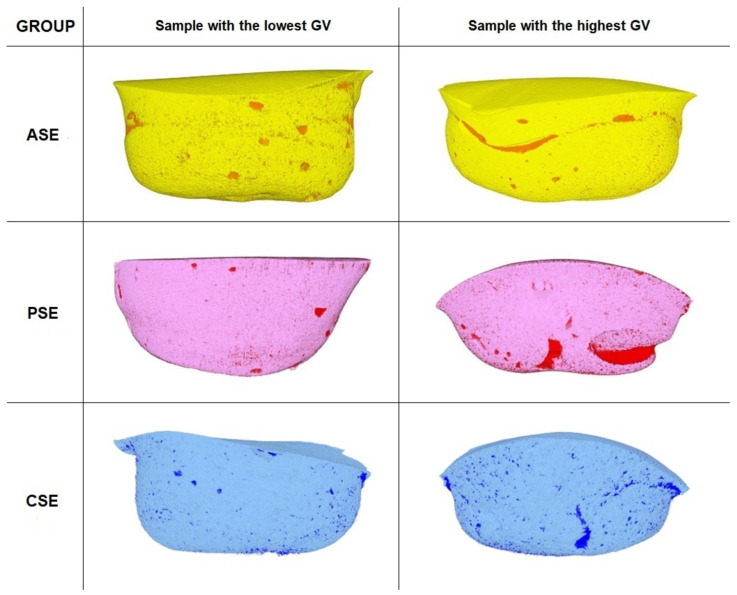
Three-dimensional reconstructions of samples with the highest and the lowest external gap volume values from the self-etch groups (free space marked by the most intense color of the reconstruction).

Three-dimensional reconstructions of samples with the highest and the lowest GV values from the etch and rinse and self-etch groups are depicted in Figure 3 and Figure 4, respectively.

### 3.2. Gap Volumes at the Tooth–Filling Interface of the External (EGV) and Internal Parts (IGV) of Cavities

The smallest EGV and IGV were measured in the AER (EGV = 0.123 mm^3^; IGV = 0.146 mm^3^) and ASE (EGV = 0.114 mm^3^; IGV = 0.101 mm^3^) groups. The biggest EGV and IGV of an etch and rinse technique were obtained for OptiBond FL (EGV = 0.279 mm^3^) and Peak Universal (IGV = 0.187 mm^3^). The biggest EGV and IGV of a self-etch technique were obtained for Clearfil SE (EGV = 0.321 mm^3^; IGV = 0.140 mm^3^).

When comparing the external and internal parts of the cavities between the experimental groups, we observed that, in the etch and rinse groups, greater gap volumes occurred in the internal part of the cavity (PER = 0.187 mm^3^; AER = 0.146 mm^3^). In contrast, for the self-etch technique, greater gap volumes occurred in the external part of the cavity (PSE= 0.189 mm^3^; ASE = 0.114 mm^3^). However, there were no statistical differences in location-dependent gap volumes (external vs. internal part) in all experimental groups.

Significant differences between the gaps in external and internal parts of the tooth–filling interface were only noted in the control groups, where significantly higher gap volumes were obtained in the external part for the CSE group (0.321 mm^3^, *p* = 0.0019) and for the OER group (0.279 mm^3^, *p* = 0.0024). The mean EGV and IGV values and standard deviations are presented in Table 3.

**Figure 5:**
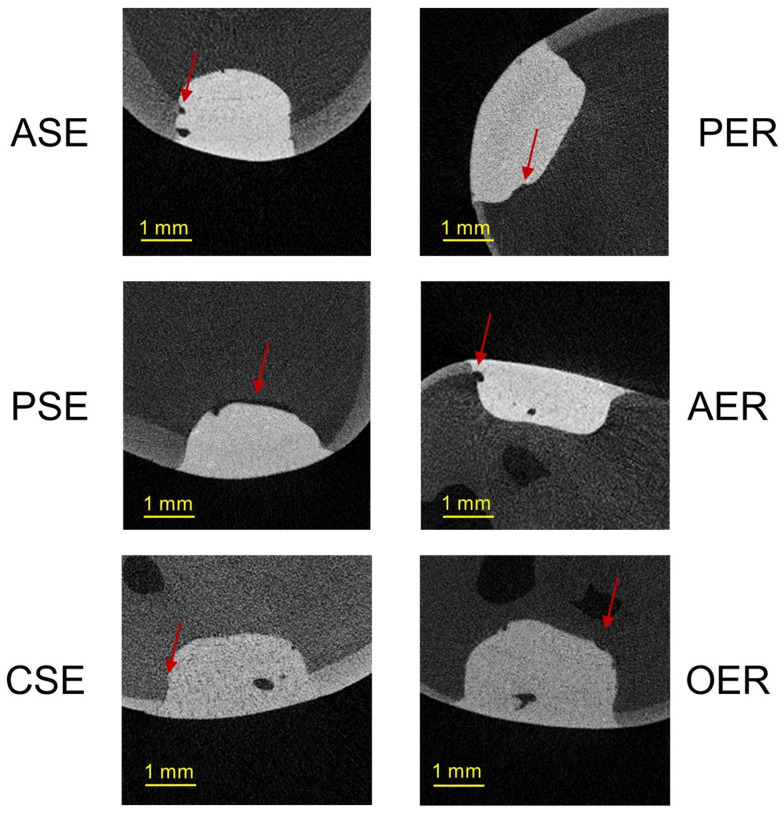
Representative µCT scans from each group (red arrows show gaps at tooth–filling interface).

Representative µCT scans from each group are presented in Figure 5.

## 4. Discussion

The immediate factors influencing gap formation at a tooth–filling interface include type of adhesive and composite resin, polymerization shrinkage and elastic modulus of filling material, configuration factors of the cavity, application method of adhesive, layering protocols of composite resin, curing methods, operator skills, and external conditions [29,30,31]. In this study, the type of cavity, application mode of composite resins, and operator skills were equal across all specimens; only the composite resin was varied according to the adhesive manufacturers’ guidelines. The cavities were filled with two layers to decrease polymerization shrinkage, the same plunger was used to fill all cavities to level the effect of the stickiness, and materials were applied in similar conditions [32]. All cavities were prepared and filled by one researcher to avoid operator variance. Moreover, in order to minimize the risk of bias, the researcher (A.K.P.) performing the µCT scans and analyses was not related to dentistry and not familiar with the materials tested, but only trained in the correct interpretation of the obtained µCT images. All doubts related to the sample analysis were discussed with the supervisor researcher (A.N.).

### 4.1. Thermomechanical Loading (TML) and Water Degradation Analysis

In this study, the specimens were artificially aged using TML and long-term water storage in distilled water. Changing temperatures cause damage to the tooth–filling interface and facilitate water degradation of exposed etched dentin areas. Moreover, mechanical loading causes tooth and filling deformations, which lead to increases in existing gaps or the development of new gaps at the tooth–filling interface [16,17,33]. Unfortunately, there is a lack of standardized TML protocols; however, the parameters used in this study were successfully implemented in previous studies [7,22,33]. Water storage is an easier and cheaper method but more time-consuming than thermocycling or mechanical loading; however, the combination of those three aging methods can reproduce the conditions in the oral cavity [34]. The most commonly used method of long-term storage is storage in water or distilled water at 37 °C for a certain time period [35]. Other incubator conditions include artificial saliva and add antibacterial solutions (e.g., sodium azide or chloramine) [17]. In previous studies, long-term storage was applied for different durations, e.g., 6 months [36,37], 12 months [38], or even 5 years [39]. In this study, the duration of 4 years of water storage was chosen according to a study by Kiyomura [40], who reported that a storage time between 2 and 4 years is required to detect the effects of hydrolytic degradation.

### 4.2. Micro-Computed Tomography (µCT) Analysis

The internal marginal adaptation of composite resin filling made with two universal adhesives was analyzed using µCT, which is an appliance used to create 3D images of small objects with a high spatial resolution. This method can be used to obtain precise information about the internal and external adaptation of materials and dental tissues without destroying the samples. However, the main disadvantages of µCT are the high cost of sample analysis, necessity of computer expertise, and large data size [41]. Although some authors consider µCT to be an effective tool for the assessment of the structure of the adhesive layer, it is not recommended, because adhesives have less radiopacity than resin composite and tooth tissues [28,42,43]. This study did not analyze the structure of the adhesive layer but focused on occurrence of the gaps (free spaces) at the tooth–filling interface, using a method of analysis similar to previous research [25,28]. The GV was calculated as the free (radiolucent) space at the tooth–filling interface without including potential voids inside the composite filling. The GV/FV and GV/CV ratios were introduced to make samples comparable between the groups despite differences in CV. To increase the reliability of the study, the actual cavity sizes were calculated based on the µCT, instead of using the initially planned dimensions. In the literature, some research use µCT and dye tracer, e.g., silver nitrate, to analyze microleakage and to find out even a small amount leakage in the tooth–filling interface [23,44] or use µCT and magnetic nanoparticles to quantitative assessments of dental adhesive layer [45]. On the other hand, the use of silver nitrate disturbs analysis in the enamel area [44].

Hirata et al. [46] and Algamaiah et al. [47] analyzed in µCT the influence of presence of adhesive layer on volumetric shrinkage of composite resins or gap development, and they noticed that the use of dental adhesive decreased the overall volumetric shrinkage [46,47] and gap development [46]. This study was focused on dental adhesive’s aspect, as the outermost layer of the filling, which is in direct contact with the tooth tissues, and it aimed to compare the gap formation of tooth–filling interfaces in NCCLs after TML and 4 years of water storage.

### 4.3. Gap Volume to Filling Volume (GV/FV) and Gap Volume to Cavity Volume (GV/CV) Ratios

Adhese Universal achieved significantly smaller GV/FV and GV/CV ratios at the tooth–filling interface compared to Peak Universal in both etching techniques (Table 2); thus, the first null hypothesis was rejected. Adhese Universal has a pH = 2.5–3 and belongs to group of “ultra-mild” adhesives, which are characterized by the formation of a “more resistant to hydrolysis” hybrid layer [48,49]. In contrast, Peak Universal has a pH = 1.2 and belongs to the group of “intermediate” adhesives [48]. Moreover, Peak Universal has chlorhexidine in its composition, which is one of the most effective matrix metalloproteinases inhibitors [50]. Other advantages of chlorhexidine addition to adhesives are antibacterial, antiproteolytic, re-wetting, and buffering properties [36]. Loguercio et al. [39] analyzed 5-year bonding properties of dentin–filling interfaces after adding 2% chlorhexidine to the acid or before applying the adhesive. They found that chlorhexidine had a positive effect on nanoleakage and the micro-tensile bond strength; however, they noticed that chlorhexidine molecules are large and water-soluble, meaning that they could leach out of the tooth–filling interface over time, causing formation or development gaps, especially in the external part of the cavity [39]. Brackett et al. [38] observed a positive effect on the dentin–filling interface after 12 months when 2% chlorhexidine was used as a primer. In conclusion, using higher concentrations of chlorhexidine before applying the adhesive could have more beneficial effects on the bonding stability than using chlorhexidine as an adhesive ingredient.

The significantly lower GV/FV and GV/CV ratios of Adhese Universal compared to those of Peak Universal in both etching techniques may result from the presence of 10-methacryloyloxydecyl dihydrogen phosphate (10-MDP) monomer in its composition. 10-MDP monomer is water-resistant and can bond with calcium from residual hydroxyapatite crystals on the dentine collagen. This chemical bond is important because it protects the adhesive–dentin interface from water degradation over time [51,52].

Adhese Universal and Peak Universal belong to the newest group of dental adhesives characterized as “universal” or “multipurpose”, which can be used in either SE or ER mode, according to the situation and operator’s preference. Universal adhesives should provide stable and long-lasting bonding regardless of the application method [2]. The ASE group had significantly lower GV/FV and GV/CV ratios than the ATE group, and the results of PSE and PER were comparable (the GV/FV and GV/CV ratios of the PSE group were not significantly lower than that of the PTE group); thus, the second null hypothesis was partially rejected. Greater gap volume at the tooth–filling interface in the ER groups compared to those in the SE groups may be due to excessive etching in the ER approach. Over-etched dentin by phosphoric acid may have prohibited the adhesive from penetrating to a sufficient depth. Consequently, free spaces that the adhesive did not reach are more prone to water degradation, which reduces bond durability. Conversely, the comparable results for both etching groups of Peak Universal may be explained by its low pH value (1.2). The pH value is closely related to the interaction depth in dentin; the lower the pH, the deeper the penetration of adhesive to dentin; thus, the risk of etched dentin not covered by adhesive is reduced [53].

The composite resin material has an important impact on the formation of gaps at the tooth–filling interface, especially while the cavity is filling, because polymerization shrinkage generates stress at the tooth–filling interface and may lead to gap formation [23]. Composite resin IPS Empress Direct (Ivoclar Vivadent; Schaan, Lichtenstein), which was used with Adhese Universal, characterized the lowest volumetric polymerization shrinkage (no information for Clearfil Majesty ES-2 (Kuraray Noritake Dental; Tokyo, Japan)), which had influence on significantly smaller GV/FV and GV/CV ratios at the tooth–filling interface compared to Peak Universal and OER groups.

### 4.4. Gap Volumes at the Tooth–Filling Interface of the External (EGV) and Internal Parts (IGV) of Cavities

This is the first study to analyze gap locations at the tooth–filling interface by dividing the cavity into an external and an internal part. Gaps in the internal part of the tooth–filling interface indicate adhesion abnormalities, especially at the pulp wall. Short- and long-term stable adhesion and hybrid layers in the internal part of the cavity should reduce the cytotoxicity of adhesives, protect from bacterial leakage, and minimize the risk of pulpal inflammation [4,5,14,15]. For the ER experimental groups, larger gap volumes were in the internal part of the cavity, whereas, for all SE groups, larger gap volumes were in the external part of the cavity, but the results were not significant in the experimental groups (Table 3); thus, the third null hypothesis was rejected. A smaller gap concentration in the external part than internal part of the cavity in both experimental ER groups may be the result of presence of the enamel in the external part of the cavity. Etching the enamel before use of universal adhesive is recommended because it improves bonding durability, and it is known as the selective etching technique [3]. Exactly evaluating the internal adaptation provides insights into the micromorphology of the tooth–filling interface and a better understanding of the existing limitations and failures in adhesive dentistry [54]. Zhao et al. [44] analyzed the leakage around class V cavities using µCT and divided the margin in cervical and coronal parts of the external tooth–filling interface for detailed analysis of microleakages. The current study and the study of Zhao et al. [44] show the additional advantage of µCT, which enables cutting the image in any direction to obtain a 3D mapping of the leakage or tooth–filling interface.

This study had some limitations. It was evaluated after TML in combination with long-term water storage. Some irregularities at the tooth–filling interface emerged while applying the adhesive and composite resin. Moreover, temperature changes combined with mechanical loading have a greater negative influence on the tooth–filling interface than water degradation. Therefore, it is recommended to perform additional µCT analysis, immediately after sample preparation and after TML, to analyze those aspects.

## 5. Conclusions

Internal gap formation and their development at the tooth–filling interface depend on factors related to the material as well as the conditions of its application. This study emphasizes the importance of adhesive compositions, which can have an influence on the long-term bonding durability. For both etching modes, ultra-mild Adhese Universal containing 10-MDP monomer provided better long-term results than both Peak Universal and current gold-standard adhesives. Moreover, pre-etching dentin should be carefully used, because it can have negative effects on the bonding stability. µCT, as used in this study, is a useful tool to analyze progressive changes in tooth fillings subjected to various aging factors.

## Figures and Tables

**Table 1:** Adhesive, etching technique, composition, and composite resin of the experimental and control groups.

Group		Adhesive (Manufacturer; Batch Number)	Etching Technique	Composition	Composite Resin (Manufacturer; Batch Number)	Type of Composite Resin; Particle Size; Filler Content (wt/vol.%); Volumetric Polymerization Shrinkage
Experimental	PER	Peak Universal (Ultradent, South Jordan, UT, USA; 5135 BBBXD)	Etch and rinse	Ethyl alcohol, HEMA, methacrylic acid, 0.2% chlorhexidine di (acetate)	Amelogen Plus (Ultradent, South Jordan, UT, USA; 9031 BB5BJ)	Microhybrid; 0.7 μm, 76/61, 2.97% [26]
	PSE		Self-etch			
	AER	Adhese Universal (Ivoclar Vivadent; Schaan, Lichtenstein; U23288)	Etch and rinse	MDP, bis-GMA, HEMA, MCAP, D3MA, ethanol, water, initiator, stabilizers, silicon dioxide	IPS Empress Direct (Ivoclar Vivadent; Schaan, Lichtenstein; T39377)	Nanohybrid; 40 nm–3 μm; 75–79/52–59, 2.13% [27]
	ASE		Self-etch			
Control	OER	OptiBond FL (Kerr, Orange, CA, USA; 5457273)	Etch and rinse	Primer: HEMA, glycerol phosphate dimethacrylate, mono-2-methacryloyloxyethyl phthalate, water, ethanol Bond: bis-GMA, HEMA, glycerol dimethacrylate, filler particles (fumed SiO_2_, barium aluminoborosilicate, Na_2_SiF_6_)	Herculite XRV Ultra (Kerr, Orange, CA, USA; 5136056)	Nanohybrid; 40 nm–30 μm; 78/59, 2.7%
	CSE	Clearfil SE (Kuraray Noritake Dental; Tokyo, Japan; 000147)	Self-etch	Primer: water, 10-MDP, HEMA, hydrophilic aliphatic dimethacrylate, accelerators, dl-camphorquinone Bond: 10-MDP, bis-GMA, HEMA, initiators, colloidal silica, dl-camphorquinone, accelerator	Clearfil Majesty ES-2 (Kuraray Noritake Dental; Tokyo, Japan; 3J0009)	Nanohybrid, 370 nm–1.5 μm, 78/66; no information

PER: Peak Universal etch and rinse; PSE: Peak Universal self-etch; AER: Adhese Universal etch and rinse; ASE: Adhese Universal self-etch; OER: OptiBond—control group for etch and rinse technique; CSE: Clearfil SE—control group for the self-etch technique.

**Table 2:** Quantified analysis obtained by µCT: GV—gap volume at tooth–filling interface; FV—filling volume; CV—cavity volume, calculated as GV + FV; GV/FV ratio (gap volume/filling volume) and GV/CV ratio (gap volume/cavity volume) are expressed as mean ± standard deviation.

Group		GV (mm^3^)	FV (mm^3^)	CV (mm^3^)	GV/FV (%)	GV/CV (%)
Etch and rinse	PER	0.369 ± 0.107	11.930 ± 1.415	12.299 ± 1.444	0.031 ± 0.009 ^de^ Min. 0.022 Max. 0.044	0.030 ± 0.008 ^DE^ Min. 0.021 Max. 0.042
	AER	0.269 ± 0.106	14.073 ± 1.777	14.342 ± 1.777	0.019 ± 0.005 ^bcdg^ Min. 0.013 Max. 0.028	0.018 ± 0.005 ^BCDG^ Min. 0.013 Max. 0.027
	OER	0.426 ± 0.049	14.688 ± 1.435	15.114 ± 1.466	0.029 ± 0.003 ^cf^ Min. 0.025 Max. 0.033	0.028 ± 0.002 ^CF^ Min. 0.024 Max. 0.032
Self-etch	PSE	0.322 ± 0.070	13.496 ± 1.795	13.818 ± 1.804	0.024 ± 0.006 ^ac^ Min. 0.016 Max. 0.040	0.024 ± 0.006 ^AC^ Min. 0.016 Max. 0.038
	ASE	0.215 ± 0.076	14.137 ± 1.972	14.352 ± 2.015	0.015 ± 0.004 ^abef^ Min. 0.010 Max. 0.026	0.015 ± 0.004 ^ABEF^ Min. 0.010 Max.0.025
	CSE	0.461 ± 0.137	14.306 ± 1.620	14.767 ± 1.708	0.032 ± 0.009 ^ag^ Min. 0.013 Max. 0.043	0.031 ± 0.008 ^AG^ Min. 0.013 Max. 0.041

The same lowercase superscript indicates a difference at the 5% significance level between GV/FV ratios. The same uppercase superscript indicates a difference at the 5% significance level between GV/CV ratios.

**Table 3:** Gap volumes at the tooth–filling interface in the external (EGV) and internal (IGV) parts of the cavity and the total gap volume at the tooth–filling interface expressed as mean ± standard deviation.

Group		EGV (mm^3^)	IGV (mm^3^)	GV (mm^3^)
Etch and rinse	PER	0.181 ± 0.069	0.187 ± 0.083	0.369 ± 0.107
	AER	0.123 ± 0.037	0.146 ± 0.078	0.269 ± 0.106
	OER	0.279 ± 0.074 *	0.147 ± 0.075 *	0.426 ± 0.049
Self-etch	PSE	0.189 ± 0.079	0.132 ± 0.087	0.322 ± 0.070
	ASE	0.114 ± 0.040	0.101 ± 0.075	0.215 ± 0.076
	CSE	0.321 ± 0.121 *	0.140 ± 0.043 *	0.461 ± 0.137

* indicates a difference at the 5% significance level between external and internal part of the cavity.

## Data Availability

Data are contained within the article.
